# Combined anterior and posterior ankle impingement syndrome with nonunion of Cedell fracture in a 58-year-old female: a case report

**DOI:** 10.1186/s12891-020-03584-9

**Published:** 2020-08-18

**Authors:** De-an Qin, Zhi-zhen Jin, Jie-fu Song

**Affiliations:** Department of Orthopedics, Shanxi Provincial People’s Hospital Affiliated to Shanxi Medical University, NO. 29, Double Tower Street, Taiyuan, 030012 Shanxi China

**Keywords:** Ankle, Anterior and posterior impingement, Fracture, Posteromedial tubercle, Talus

## Abstract

**Background:**

Combined anterior and posterior ankle impingement has seldom been reported. Cedell fracture, fracture of posteromedial tubercle of talus, is an uncommon and easily missed injury which may elicit posteromedial ankle impingement. The injury mechanisms and management strategies of these two lesions have been reported individually. But the concurrent lesion of both of them has not been reported.

**Case presentation:**

We reported a 58-year-old female with combined anterior and posterior ankle impingement syndrome with nonunion of Cedell fracture in whom open osteophytes debridement, fracture internal fixation and posterior talotibial ligament reconstruction were performed. The AOFAS hindfoot score was 90 at 1 year follow-up. To our knowledge, this was the first reported case with anterior, posterior and posteromedial impingement which was treated operatively with an excellent short-term outcome.

**Conclusions:**

To fully recognize this occult lesion and avoid missing is imperative for reducing the morbidities. We suggest CT and MRI as excellent imaging modalities that can help the timely diagnosis and appropriate treatment for this combined impingement with circumferential lesions.

## Background

Fracture of posteromedial tubercle of talus (PMTT), first described by Cedell in 1974 [1], is an uncommon injury caused by dorsiflexion-pronation of the foot and ankle as the posterior talotibial ligament is torn from its attachment to the talus. The early detection of this fracture is difficult [2]. The displaced, old or nonunited fracture can compress the surrounding structures and elicit the posteromedial impingement syndrome which will require subsequent surgical excision or internal fixation of the fragment [3]. Anterior and posterior ankle impingement are well documented in the literatures, but are generally considered as separate entities with different injury mechanisms. Combined anterior and posterior ankle impingement has seldom been previously reported [4]. The concurrent lesion of both the combined anterior and posterior ankle impingement and the nonunited Cedell fracture has not been reported. We reported a unique case of a 58-year-old female with combined anterior and posterior ankle impingement syndrome with nonunion of Cedell fracture. We performed open osteophytes debridement, fracture internal fixation and posterior talotibial ligament reconstruction. To recognize this occult lesion and avoid missing is imperative for reducing the morbidities.

## Case presentation

A 58-year-old female gymnastics teacher presented to our orthopedic clinic with painful and swollen right ankle for 3 months. She twisted her right ankle during a game 3 months before in ankle dorsiflexion position. She was initially seen at a local emergency room. Plain radiographs for her right ankle showed the degenerative changes. She was treated with cast immobilization and rest. But swelling, pain and movement limitation persisted. She had a history of right ankle sprain 5 years before and since then she sustained intermittent ankle pain and swelling especially under undue exercise. On examination, she had a noticeable limp and tried to reduce her body weightbearing on her right ankle. There were deep localized tenderness and swelling on anterior ankle, posterior ankle, and behind the medial malleolus individually. Tenderness could be exacerbated by extreme passive ankle dorsiflexion and plantarflexion. The impingement provocative tests were positive. There was a 1-cm diameter firm, tender mass posteroinferior to the medial malleolus. Her right ankle exhibited a decreased dorsiflexion of 10° and a mild valgus instability compared with the contralateral. Circulation, sensation, and motor of her toes and hallux were intact.

**Fig. 1:**
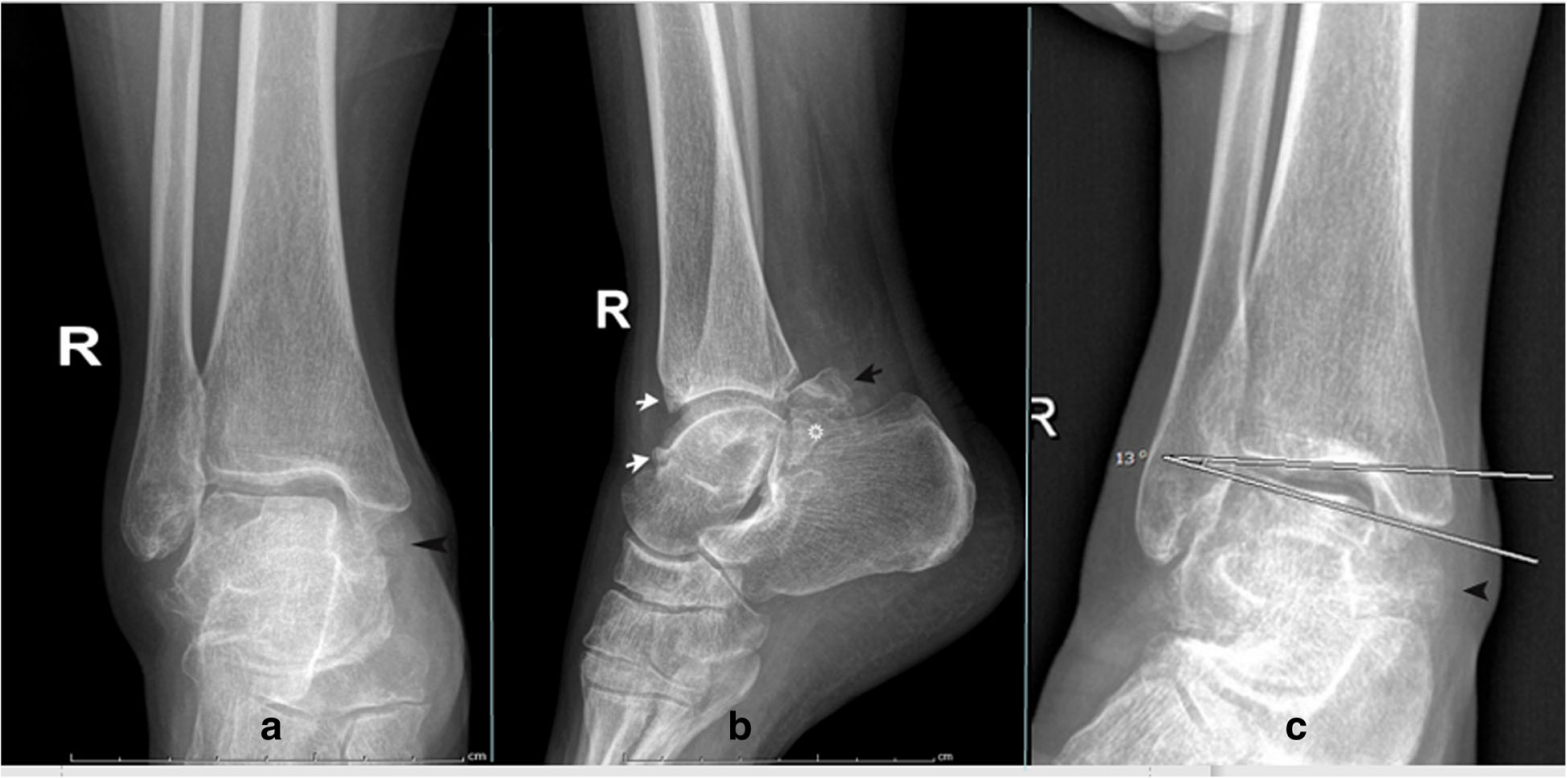
The radiographs of right ankle. **a** Anteroposterior weight-bearing view showing abundant osteophytes below the medial malleolus (arrowhead).**b** Lateral weight-bearing view showing osteophytes on the dorsal talar neck (white short arrow), anterior tibial plafond (white short arrow), posterior talar process (black short arrow), and the PMTT fracture fragment (star) with preservation of the tibiotalar joint space. c, Valgus stress view showing a talar tilt of 13° angle

**Fig. 2:**
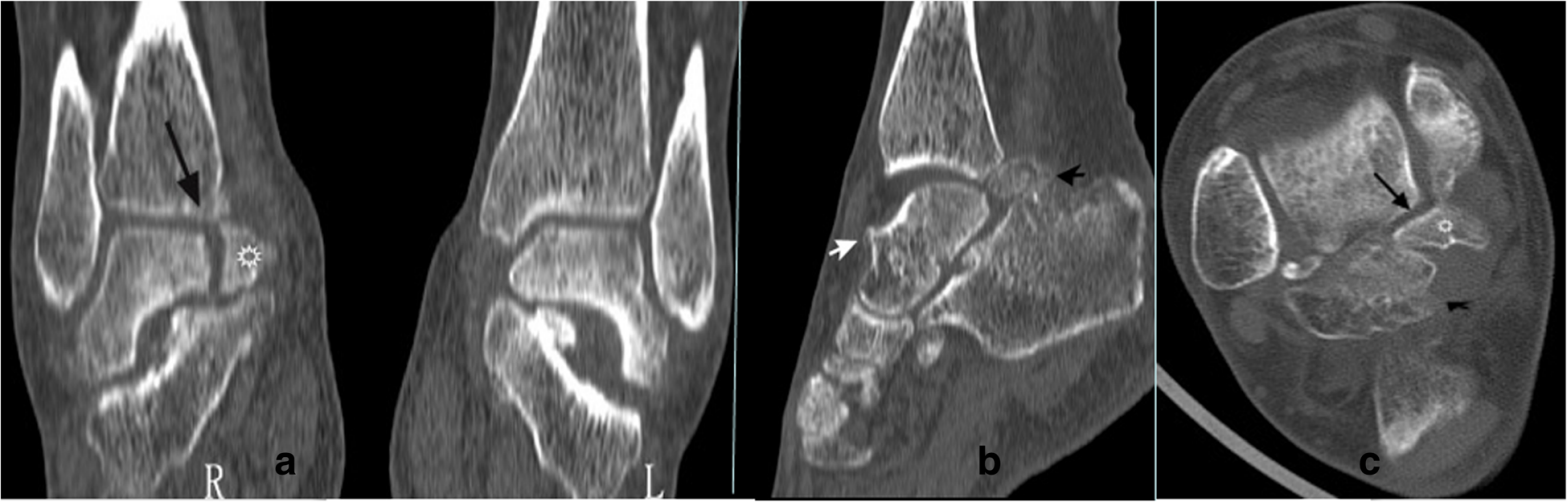
CT scans. **a** coronal view showing a nondisplaced, nonunited PMTT fracture fragment (star) and an interface between the fracture fragment and the parent talus (long arrow). **b** sagittal view showing the anterior and posterior osteophytes of impingement (white and black short arrow). **c** axial view showing the larger posterolateral tubercle osteophyte (black short arrow), the nonunited PMTT fragment (star) and the interface (long arrow)

**Fig. 3:**
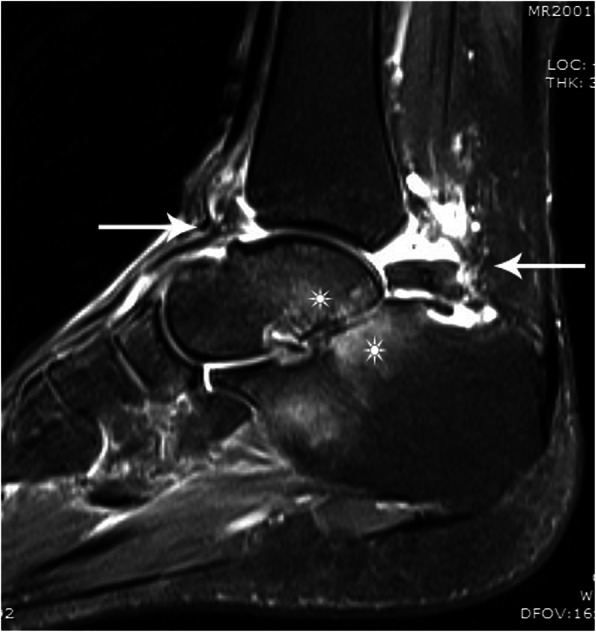
MRI sagittal PDWI showing the anterior, posterior ankle joint effusion (long arrow) and the “Kissing contusions (sun)” on the posterior aspect of the posterior subtalar articulation

Anteroposterior weight-bearing radiograph for right ankle showed osteophytes below the medial malleolus (Fig. 1a). Lateral weight-bearing radiograph showed osteophytes on the dorsal talar neck, anterior tibial plafond, posterior talar process, and the PMTT fracture fragment with preservation of the tibiotalar joint space (Fig. 1b). Valgus stress radiograph showed a talar tilt of 13° angle (Fig. 1c). 64-detector computed tomography (CT) coronal view showed a nondisplaced, nonunited PMTT fracture fragment and an interface between the fracture fragment and the parent talus (Fig. 2a). The sagittal view showed the anterior and posterior osteophytes of impingement (Fig. 2b). The axial view showed the larger posterolateral tubercle osteophyte, the nonunited PMTT fragment and the interface (Fig. 2c). 3.0-Tesla magnetic resonance imaging (MRI) sagittal proton density weighted image (PDWI) showed the anterior and posterior ankle joint effusion. The “Kissing contusions”, traumatic bone marrow edema on the posterior aspect of the posterior subtalar articulation was also apparent (Fig. 3).

A diagnosis of combined anterior and posterior ankle impingement syndrome with nonunion of Cedell fracture was made. Considering her chronic persistent pain, she was suggested surgical intervention.

**Fig. 4:**
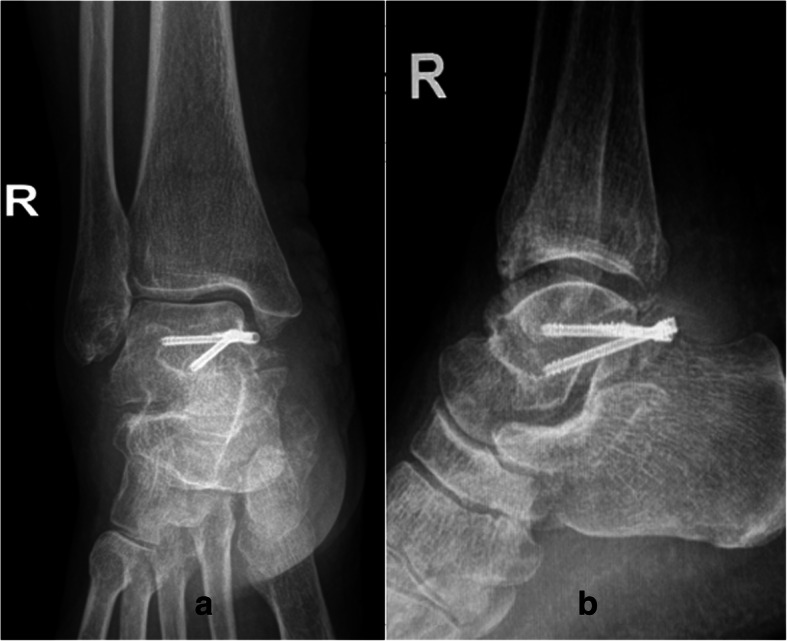
Postoperative radiographs. **a** anteroposterior and **b** lateral view showing the osteophyte debridement and screw position

In view of the large extent of osteophytes and old fracture, we did not attempt arthroscopic portals and decided an open surgical intervention. She was given general anesthesia and placed in a supine position with a thigh tourniquet inflated to 300 mmHg. A 6 cm posteromedial curvilinear incision was made just posterior to the medial malleolus and over the bony prominence. After incision of the flexor retinaculum, dissection proceeded between the posterior tibialis tendon and flexor digitorum longus tendons anteriorly and the posterior tibial artery, tibial nerve, and flexor hallucis longus (FHL) tendon posteriorly. After ankle plantarflexion and posterior retraction of FHL, the nonunited fracture fragment of PMTT was exposed. Posterior ankle osteophyte which elicited posterior impingement could be found behind the PMTT fragment and was debrided. The fracture section of talus was scraped fresh with a curette. The PMTT fracture fragment was reduced under direct visualization, and internal fixation was performed using two 3.0-mm cannulated Herbert screws (Zimmer, Warsaw, USA) with perpendicular to the fracture section. A 4 cm ankle anteromedial longitudinal incision was made and the tibial anterior tendon was retracted laterally. This incision was not at the same level as the posteromedial incision. The arthrotomy was performed along the distal tibial epiphysis and talar neck. The ankle joint cavity was probed and no free bodies or talar osteochondral lesions were found. The anterior tibial plafond osteophyte was removed by rongeur. The talar neck osteophyte was resected with small osteotome. Intraoperative fluoroscopy was used to verify osteophyte debridement and screw position. The ankle valgus stress test was performed and showed the medial instability. The posterior talotibial ligament was repaired with a suture anchor (Smith & Nephew, Memphis, TN, USA) to the PMTT in the medial-to-lateral direction. The retinaculum was repaired and the skin was closed. A short leg cast was applied for 6 weeks in an ankle neutral position and partial weightbearing began after 6 weeks. Full weightbearing was allowed when radiographic union was achieved at 12 weeks postoperatively. Postoperative radiographs showed the osteophyte debridement and screw position (Fig. 4a, b). The American Orthopedic Foot and Ankle Society (AOFAS) hindfoot score was 90 at 1 year follow-up.

## Discussion and conclusion

Fracture of the PMTT is an uncommon injury with significant morbidities such as tarsal tunnel syndrome [5], FHL tendon interposition [6, 7], posteromedial ankle impingement [3, 8]. The posterior process of talus is composed of medial and lateral tubercles, with an intervening groove for passage of the FHL tendon. Fracture of the larger posterolateral tubercle is caused by inversion or extreme equinus and was first described by Shepherd in 1882, an ossicle known as the os trigonum contributing to the posterior ankle impingement. Fracture of the smaller posteromedial tubercle is caused by excessive dorsiflexion and pronation and was first described by Cedell in 1974, a posterior talotibial ligament avulsion fracture contributing to the posteromedial impingement. Approximately 25% of the posterior articular facet of the subtalar joint is covered by the talar posterior process; hence, timely diagnosis and appropriate treatment is of utmost importance to prevent subtalar arthritis. The Cedell fracture is easily misdiagnosed as ankle sprain due to its rarity and anatomic location for superimposition projections on plain anteroposterior and lateral radiographs. Oblique view radiograph, in which the foot is 30°externally rotated, can be used if this injury is suspected [9, 10]. MRI and CT can be helpful in ascertaining the diagnosis, determining the size of the fracture fragment and degree of displacement. Also, in the case of a chronic injury, the presence of fibrous union, ligamentous disruption, and traumatic edema can be showed. The importance of using these advanced imaging studies cannot be overemphasized when fracture or nonunion of the PMTT is suspected [11].

Different management strategies for Cedell fracture are reported in the literatures: conservative treatment of nondisplaced or minimally displaced fractures [2], open reduction internal fixation for displaced fractures [6], or fracture excision for malunion of displaced fractures by open or arthroscopy [9, 12]. Watanabe et al. [2] classified the fracture of PMTT into 3 types according to the mechanism of injury and the fracture configuration: Avulsion type, Split type and Comminuted type. Conservative treatment by cast immobilization is recommended for the split-type or nondisplaced, fresh avulsion-type fractures. Bone excision is recommended for the displaced or nondisplaced, but old, nonunited avulsion-type fractures, because in these cases, the fractured fragment might cause posteromedial ankle impingement. Open reduction and internal fixation is recommended for the comminuted-type fractures. There are also data to support improved outcomes with early excision of larger avulsion fractures of the PMTT. But Shank et al. [8] thought most peripheral talus fractures involve sizable articular components with displacement of the ankle, subtalar, and talonavicular joints and suggested open reduction internal fixation for improving efficacy and delaying arthrosis. Conservative treatment or excision should be reserved for smaller avulsion fractures or severely comminuted fractures that are not amendable to open reduction internal fixation. Excision or primary arthrodesis should be used as a last-resort option.

Ankle impingement syndromes are common disorders and may be due to interposition of soft or osseous tissues. The latter is the most common cause [13]. According to location, ankle impingement can be classified as anterolateral, anterior, anteromedial, posteromedial, and posterior. Anterior or posterior impingement is common; however, combined anterior and posterior ankle impingement syndrome is a novel lesion resulting from a specific type of injury, instead of a simple combination of these two [14]. The etiology is complex and may be repetitive inversion injury. The diagnosis is based on the consistent clinical symptoms, physical examinations and imaging findings. Radiography may show anterior spurs arising from anterior tibial plafond or/and dorsal talar neck, combining with posterior tibial plafond, elongated posterior talar process, or os trigonum [15]. CT can best demonstrate the spurs or small cortical avulsion fractures. MRI can demonstrate other associated signs of symptomatic impingement, including marrow edema at the site of spur formation, ligament injury, joint effusion, or synovial thickening, et al. [16].

A review of the published data reveals that few studies have been reported regarding the treatment of combined anterior and posterior ankle impingement. The disorder can be treated arthroscopically or by open debridement, both with good outcomes [17]. Henderson et al. [18] treated 62 patients using anterior arthroscopy and posterior open arthrotomy with the patients in a supine position. Kim et al. [19] treated 24 patients with anterior ankle arthroscopy and hindfoot endoscopy. Song et al. [14] treated 28 patients by arthroscopy.

For our patient, arthroscopic debridement is challenging considering the relatively large posterior bony spur, which may require enlargement of the portal, further risking the neurovascular structure [20] [18]. Secondly, the nonunion of Cedell fracture should be fixed in view of the large extent of fragment causing the posteromedial pain and posttraumatic subtalar arthritis. Thirdly, the chronic medial ankle instability should be simultaneously managed [21]. Thus, we performed the open surgery for the patient. Besides osteophytes debridement, we fixed the PMTT fracture fragment with two Herbert screws in different direction of perpendicular to the fracture section and achieved interfragmentary compression. The limitation of our case is short-time report and long-term outcome needs further follow-up.

We described a unique case of combined anterior and posterior ankle impingement syndrome with nonunion of Cedell fracture and reviewed the diagnosis and management strategies. We suggest CT and MRI as excellent imaging modalities which can help the timely diagnosis and appropriate treatment for this combined impingement with circumferential lesions.

## Data Availability

The datasets used and/or analyzed during the current study are available from the corresponding author on reasonable request.

## Abbreviations

PMTT: Posteromedial tubercle of talus
CT: Computed tomography
MRI: Magnetic resonance imaging
FHL: Flexor hallucis longus
